# Possible melatonin-induced salt stress tolerance pathway in *Phaseolus vulgaris* L. using transcriptomic and metabolomic analyses

**DOI:** 10.1186/s12870-023-04705-x

**Published:** 2024-01-25

**Authors:** Xiaoxu Yang, Dajun Liu, Chang Liu, Mengdi Li, Zhishan Yan, Yu Zhang, Guojun Feng

**Affiliations:** 1 Horticulture Department, College of Advanced Agriculture and Ecological Environment, Heilongjiang University, 74 Xuefu Road, Harbin, Heilongjiang 150000 China; 2 Jiaxiang Industrial Technology Research Institute of Heilongjiang University, Jining, Shandong 272400 China

**Keywords:** Common bean, Salt stress, Melatonin, Transcriptome, Metabolome

## Abstract

Melatonin plays important roles in multiple stress responses; however, the downstream signaling pathway and molecular mechanism remain unclear. This study aimed to elucidate the transcriptional regulation of melatonin-induced salt stress tolerance in *Phaseolus vulgaris* L. and identify the key downstream transcription factors of melatonin through transcriptomic and metabolomic analyses. The melatonin-induced transcriptional network of hormones, transcription factors, and functional genes was established under both control and stress conditions. Among these, eight candidate transcription factors were identified via gene ontology (GO) and Kyoto Encyclopedia of Genes and Genomes (KEGG) analyses, one gene related to transmembrane transport of salts (*Phvul.004G177300*). These genes may play a role in maintaining the cell structure and excreting sodium ions outside the cell or transporting them to the vacuoles for storage. Melatonin regulates the Phvul.009G210332 gene and metabolites C05642 (N-acetyl-N-2-formyl-5-methoxycanurine), C05643 (6-hydroxymelatonin), C05660 (5-methoxyindoleacetic acid) involved in tryptophan metabolism. The metabolites C05642 and C05643 were identified as decomposition products of tryptophan, indicating that exogenous melatonin entered the *P. vulgaris* tissue and was metabolized. Melatonin promotes the synthesis and metabolism of tryptophan, which is crucial to plant metabolism, growth, maintenance, and repair.

**Supplementary Information:**

The online version contains supplementary material available at 10.1186/s12870-023-04705-x.

## Introduction

Salinized land (9.209 million hm2) accounts for approximately one-tenth of the cultivated land in China. In recent decades, increased consumer demand for out-of-season vegetables has led to the rapid expansion of vegetable cultivation areas. In addition, specific mulching and fertilizer applications [1] have significantly altered the physical and chemical properties of the soil, together with increasing the degree of secondary salinization of the soil, severely affecting both crop yield and quality [2]. The common bean is glycophytic and shows strong sensitivity to salt, with soil salt levels of 2 dS·m − 1 and below adversely affecting crop production [3]. Pod yields were found to be reduced by 85% in soils containing 100 mM NaCl [4], although variation was seen with different cultivars, with some showing higher levels of salt tolerance [5–7]. Climate change is likely to exacerbate the problem, particularly in semi-arid and arid areas, adversely affecting subsistence farming, especially in developing countries.

Over time and as the salt concentration increases, two primary impairments take place in distinct phases: osmotic stress and specific ion toxicity. These impairments subsequently give rise to secondary stresses like oxidative stress and nutritional disorders. These effects result in decreased water and nutrient absorption, impaired membrane function, and disruption of crucial processes like respiration, photosynthesis, and protein synthesis [8–10]. Plants have the capacity to perceive stimuli from their environment and regulate defense pathways through various regulatory networks to cope with abiotic stresses, including salt stress. Salt tolerance refers to the plant’s genotype’ s ability to mitigate the harmful effects of salt by alleviating osmotic stress, ion toxicity, and the consequent oxidative stress, ultimately reducing yield losses [11, 12].

Transcriptome sequencing provides useful information on transcriptional regulation due to its significant accuracy and wide coverage, and is thus widely used in medicine and agriculture. It is often used in the study of plant biotic and abiotic stresses in agronomy research [13, 14]. This has shown high expression of PvB3 genes in rapidly growing bean tissues, including pods, nodules, and leaves. The PvB3-001/010/027/032/047/048/059/090 genes were found to be especially associated with tryprophan metabolic pathways, together with the auxin-associated genes GH3, AUX/IAA, and SAUR, after exposure of the plant to high salinity. Exposure to exogenous auxin (IAA) increased both lateral root numbers and endogenous IAA levels, together with affecting PvB3 expression. These findings suggest the involvement of PvB3s in regulating IAA metabolism under salt stress conditions in the common bean [15]. Wild barley was found to be less vulnerable to high salinity in comparison to cultivated barley, suggesting efficient responses to stress, and showed 6,048 differentially expressed genes (DEGs), of which 3,025 were up-regulated and 3,023 down-regulated after exposure to high salinity. The expression of genes responsive to salt stress was markedly reduced in salt-sensitive barley, relative to the tolerant variety, which showed 2,610 DEGs, with 580 up-regulated and 2,030 down-regulated. Salt-tolerant plants were found to express more genes associated with stress responses relative to sensitive plants [16].

In cotton plants, the key gene families involved in abiotic stress regulation, such as *WRKY*, *ERF*, and *JAZ*, were differentially expressed under salt stress [17]. Studies on barley showed that ethylene production, signaling network, and protein refolding pathways were significantly enriched under salt stress [18]. Tool in the study of plant metabolism and microbial metabolism and is increasingly being used to explore the mechanism of plant stress tolerance; the identification of associated metabolites can better elucidate the regulatory mechanism of the coping mechanism of plants under stress. Previous studies have used metabolomics to investigate the stress response of plants including barley [19, 20] and alfalfa [21]. However, single omics analysis methods have limitations; therefore, integrated multi-omics analysis is used. Previous studies have used integrated transcriptomic and metabolomic analysis to examine abiotic stress in plants. It was found that isoflavones were differentially expressed under drought stress in soybean and isoflavones played an important role in the regulation of ROS [22]. In tobacco, it was reported that increased polyphenol content may protect against low temperature stress [23]. The higher salt tolerance of sea barley a halophyte than that of common barley was found to be related to superior Na/K homeostasis, energy-saving strategies, and osmotic regulation in tissues [24]. However, to our knowledge, there are no transcriptomic and metabolomic studies of salt stress tolerance in common bean (*Phaseolus vulgaris* L.).

## Materials and methods

### Plant material and phenotypic analysis

In total, 120 common bean varieties were germinated and then sown in vermiculite in Harbin (North-East China; latitude 44°30′24″N; longitude 125°42′41″E) on May 21, 2020 (Additional file 1). Plants were cultivated in an artificial climate chamber (chamber temperature 22–25 ℃/15–18 ℃ [day/night], relative humidity approximately 75%, photoperiod 10:14 h [light: dark], irradiation intensity 800 µmol m^− 2^ s^− 1^; and CO_2_ concentration 400 µmol mol^− 1^). After the emergence of trifoliate leaves, seedlings were selected and transferred into plastic containers containing 5 L of Hoagland nutrient solution [25]. A water tank with a length of 100 cm, a width of 50 cm and a height of 10 cm was used to oxygenate the culture medium with an oxygen pump. All containers were painted with black paint to protect the exogenous melatonin and roots from light exposure. The treatments were started at 12 days after precultivation. To obtain samples for transcriptomics and metabolomics, three treatment groups were established: (1) control (marked as -C), common bean plants cultivated with only Hoagland nutrient solution; (2) stress (marked as -S), Hoagland nutrient solution plus 150 mmol·L^− 1^ NaCl; (3) stress with melatonin (marked as -SM) Hoagland nutrient solution plus 150 mmol·L^− 1^ NaCl and 100 µmol·L^− 1^ melatonin. The experiment was performed in a randomized complete block design with six replicates, and the solution was replaced every 2 days.

We use the formula (W2-W1)/W1 to calculate relative growth, relative plant height, relative leaf area, relative net photosynthesis. Relative growth (W1: Fresh quality of whole plant after 1 day, W2: Fresh quality of whole plant after 10 days), relative plant height (W1: The length whole of plant after 1 day, W2: The length of whole plant after 10 days), relative leaf area (W1: leaf area after 1 day, W2: leaf area after 10 day), relative net photosynthesis, (W1: net photosynthesis 1 day, W2: net photosynthesis 10 days), relative root–shoot ratio (Underground dry weight/above ground dry weight) and variable coefficients ((Standard deviation/Mean)×100%) were determined in this study (all with three independent biological replicates). All the growth indicators were measured before treatment and at 10 days after salt stress treatment, respectively. To determine fresh weight, three plants were randomly selected; the fresh weight of the above ground and underground parts of the whole plant was measured using an electronic balance. Plant height was measured with a meter stick, and photosynthetic pigment levels [26] and photosynthetic parameters were measured using the Li-6400 portable photosynthetic apparatus (Li-Cor Inc., USA), using six replicates per treatment. The area of the first trifoliate leaf was determined using IC Measure 2.0.0 133 software.

### Evaluation of antioxidative enzymatic activity and malondialdehyde levels

We assessed antioxidative enzymatic activity and Malondialdehyde (MDA) levels every 6 h following 10 days of salt stress. To isolate antioxidative enzymes, we homogenized 0.5 g leaves in 50 mM Na_2_HPO_4_–NaH_2_PO_4_ buffer (pH 7.8) plus 0.2 mM EDTA and 2% (w/v) insoluble polyvinylpyrrolidone, before centrifuging for 20-min at 12 000 × *g* to yield the supernatant from which enzymatic activity was analyzed. The aforementioned protocol was conducted in 0–4 ℃, and all spectrophotometric analyses employed a UV-vis sensor (UV-2401, Shimadzu Co. Ltd., Japan).

Superoxide dismutase (SOD) activity represented its performance in suppressing nitro blue tetrazolium (NBT) photochemical reduction, and utilized the Giannopolitis and Ries technique [27]. One SOD unit represented the needed enzyme quantity to induce 50% suppresstion of NBT reduction rate, as detected at 560 nm. Peroxidase (POD) activity was determined using a previously published protocol [28]. In short, we prepared a reaction mixture with 3 mL phosphate buffer (pH 7), 1 mL H_2_O_2_ (0.18%), 1 mL enzyme extract, and 1 mL guaiacol (0.1%), and the activity was assessed based on an absorbance rise from 470 nm. The O_2_^−^ synthetic rate was assessed according to the Elstner and Heupel protocol [29], employing sulfanilamide, absorbance measurement at 530 nm, and a standard NaNO_2_ curve.

MDA levels were evaluated via a slightly modified thiobarbituric acid reaction technique [30]. In short, we homogenized 0.5 g leaf samples in 5 mL 10% TCA, before centrifuging 20-min at 12 000 × *g*, mixing of supernatant with 2 mL 0.6% thiobarbituric acid (TBA), followed by a 30-min water bath heating at 100 ℃, and rapid cooking. Then, the mixture underwent 10-min centrifugation at 3000 × *g*, prior to optical density measurement at 450, 532, and 600 nm, respectively. The formula for MDA level determination is described below:

$${\rm{C }}\left( {{\rm{\mu m }}{{\rm{L}}^{{\rm{ - 1}}}}} \right){\rm{ = 6}}{\rm{.45 }}\left( {{\rm{A532 }} - {\rm{ A600}}} \right){\rm{ }} - {\rm{ 0}}{\rm{.56 \times A450}}$$

### Metabolite profiling using an ultra-performance liquid chromatography (UPLC) coupled with quadrupole time-of-flight (Q-TOF) tandem mass spectrometry (MS)

We generated 30 distinct metabolome libraries (6 separate replicates with individual sampling duration) for individual intervention category, namely, Heihu-C, Heihu-S, F4226-C, F4226-S, and F4226-SM. Firstly, the samples were placed on ice to thaw, then 50% methanol buffer was used for metabolite extraction. Briefly, we combined 20 µL sample with 120 µL precooled 50% methanol, and mixed for 1-min using vortex. We maintained the mixture at room temperature for 10 min, then at − 20 °C overnight, before centrifuging at 4000 × *g* for 20 min, prior to supernatant transfer to 96-well plates, and storage at − 80 °C until LC-MS assessment. Additionally, we collected QC samples by integrating 10 µL of all isolation mixtures using UPLC, Q-TOF, and 5600 plus tandem MS (SCIEX, UK). Subsequently, samples were maintained at − 80 °C before LC-MS analysis. Q-TOF employed both positive (PIM) and negative ion modes (NIM), with curtain gas at 30 PSI, ion source gas (ISG) 1 at 60 PSI, ISG 2 at 60 PSI, and interface heater temperature at 650 °C. We also adjusted the PIM and NIM IonSpray Voltage Floating to 5000 V and − 4500 V, respectively.

The software of SIMCA-P + 12.0 was employed to sum-normalize and Pareto-scale individual sample mass peak intensity values (Umetrics, Umea, Sweden). We next compared metabolic compositional differences among interventions using principal component (PCA) and orthogonal partial least squares discriminant analyses (OPLS-DA) of 30 samples (5 sample × 6 replicates). The metabolite reliability correlation [p(corr)] values were retrieved from the OPLS-DA S-plot with the first component.

### RNA-seq and functional annotation

Trifoliate leaves were collected to establish three biological replicates; each replicate sample (20 g) was collected from 2 to 3 randomly selected plants) at 10 days after salt stress, immediately frozen in liquid nitrogen, and stored at − 80 °C. A total of 15 RNA libraries (three independent biological replicates per sampling time) were constructed per treatment group: Heihu-C, Heihu-S, F4226-C, F4226-S, and F4226-SM.

We isolated leaf total RNA using TRIzol® (Invitrogen, CA, USA) and associated directions. After purifying, mRNA underwent divalent cation-based fragmentation, followed by reverse-transcription using mRNA-Seq sample preparation kit (Illumina, San Diego, CA, USA) to yield cDNA library. The average paired-end library insert size was 300 bp (± 50 bp), and the paired-end sequencing employed an Illumina HiSeq 4000 platform and associated directions. We performed three technical replicates for individual salt exposures to yield reliable RNA sequencing results. BLAST (2.2.3) and BLAST2GO (2.2.5) were utilized for DEG functional annotation, whereas, gene ontology (GO), and Kyoto Encyclopedia of Genes and Genomes (KEGG) were employed for signaling network enrichment analyses [31–33].

We used the HISAT package (https://jgi.doe.gov/) to align sample reads to the Phaseolus database (https://phytozome.jgi.doe.gov/pz/portal.html#!info?alias=Org_Pvulgaris). We removed read segments, based on associated read quality data, prior to plotting the residual reads to the RG. Conduct several alignments per read (< 20 by default), with 2 maximum mismatches by using HISAT. Subsequently, it generates a possible splice junction database, and verified the data via comparison of prior unmapped reads to the putative junction database.

Furthermore, we employed StringTie (2.0) to conduct mRNA content and differential regulation by assembling the mapped reads of individual samples. Next, using Perl scripts, we integrated all transcriptome assemblies from the same sample to regenerate an extensive sample-specific transcriptome. Subsequently, StringTieand edgeR were employed for mRNA content estimation. Using StringTie, we calculated Fragments Per Kilobase of exon model per Million mapped fragments (FPKM) to yield transcript expression. Significantly regulated transcripts and genes were considered to exhibit log2 (fold change) > 1 or log2 (fold change) < − 1, with *p* value < 0.05. To conduct RNA-seq assessment, we utilized PCA analysis to compute correlation coefficients (CC) (both using R 3.4.4) among samples to assess precision of our results. The CCs were based out of FPKM levels.

### Unifying assessment of metabolomic and transcriptomic information

We computed pearson correlation coefficients for the integrated metabolome and transcriptome information using the average replicate information for individual intervention in the metabolome data as well as the average mRNA content information in the transcriptome data.

## Results

### Analysis of salt tolerance of common bean core germplasm resources

In total, 120 core germplasm resources of common bean were selected as experimental materials; the seedlings were treated with 150 mmol·L^− 1^ NaCl, and the relative root–shoot ratio, relative growth, relative plant height, relative leaf area, relative net photosynthesis, and variable coefficients were determined after 10 days (Additional file 2). The results showed that the growth indexes of different varieties showed differences after salt stress. To further assess the tolerance of each variety to salt stress, the membership function and correlation analysis of each index was carried out. According to the weight analysis of the membership function (the value of the membership function reflects the impact degree of each index under salt stress) (Additional file 3). The correlation between each index and the D value was also analyzed, and the results showed that all indexes except relative plant height were positively correlated with the D value, indicating that all indexes except relative plant height were closely associated with salt tolerance in common bean (Additional file 4). On the basis of the index analysis, highly salt-tolerant varieties Heihu (P19023), Heizhenzhu (P18009), Jidou (P18012), and Jifeng (P18051) and highly salt-sensitive varieties A18, F4226 (P16028), GZ77 (P16333), and Zijiadou (P19030) were identified.

**Fig. 1:**
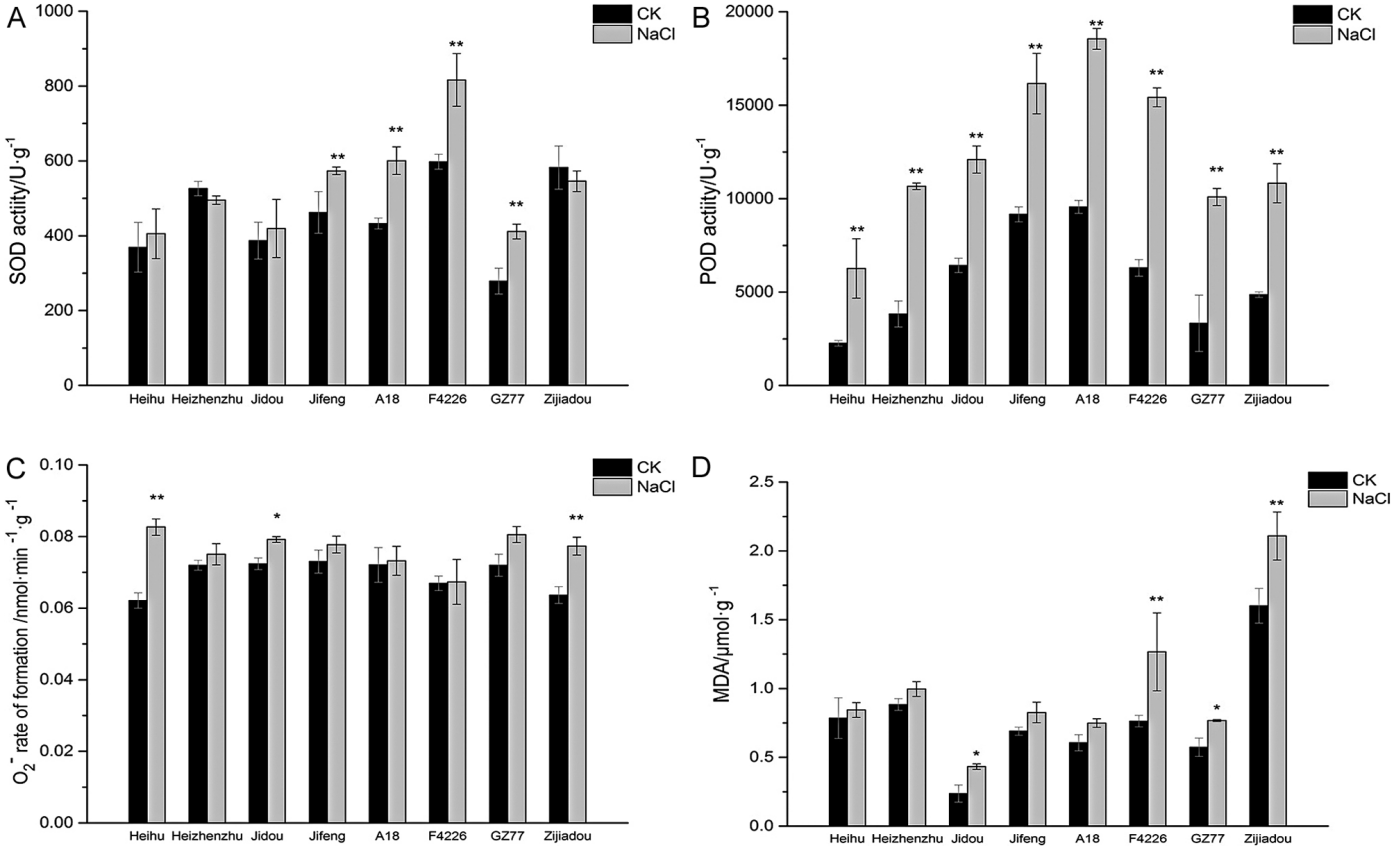
Histograms showing the observed variation for four indices in 8 bean cultivars. *Note*: (**A**) SOD, (**B**) POD, (**C**) O_2_^−^, (**D**) MDA

To further assess the varieties with high salt tolerance and salt sensitivity, the 8 identified varieties were subjected to salt stress with 150 mmol·L^− 1^ NaCl. Biochemical analysis at 10 days after salt stress treatment showed that the SOD activity of four varieties was higher in the treatment group than in the control group. Of these, F4226 showed the greatest difference (36.68%). For all varieties, the POD activity was higher in the salt stress treatment group than in the control group, with F4226 showing the greatest difference (144.99%). For some varieties, the O_2_^−^ production rate was significantly higher in the salt stress group than in the control, with Heihu showing the greatest difference (33.26%); however, for F4226, the O_2_^−^ production rate showed almost no difference between the two groups. The MDA content of Heihu under salt stress was not significantly different from that in the control group, whereas the MDA content of F4226 was significantly higher under salt stress. On the basis of this comprehensive biochemical analysis, Heihu (highly salt-tolerant variety) and F4226 (salt-sensitive variety) were used as experimental materials in further experiments (Fig. 1).

### Dynamic analysis of physiological indexes of common bean under salt stress

**Fig. 2:**
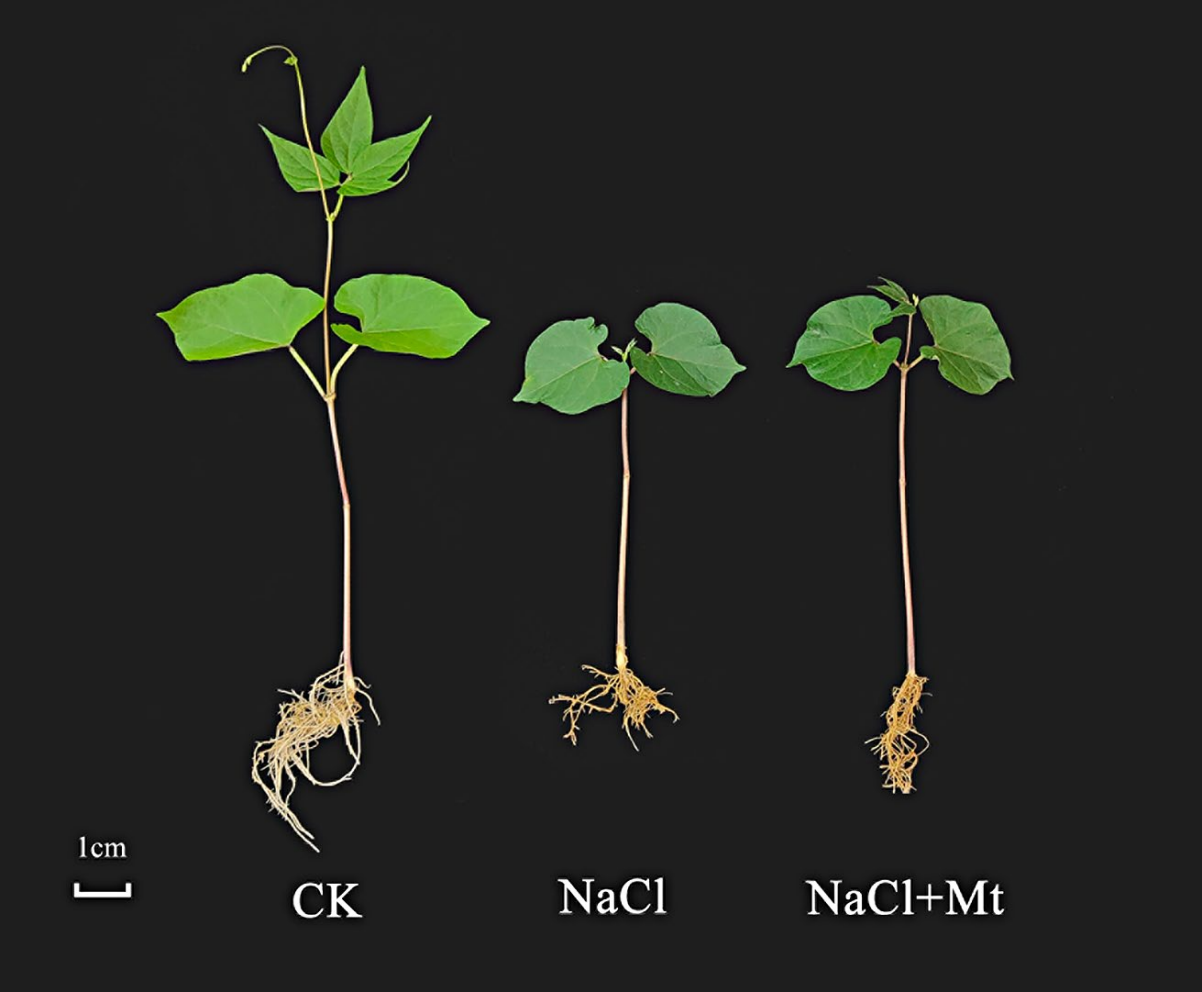
Representative phenotypes of NaCl stress mitigation by exogenous melatonin in Heihu seedlings after 10 days of salt stress treatment. *Note*: (1) CK: common bean plants cultivated with only Hoagland nutrient solution; (2) NaCl: Hoagland nutrient solution plus 150 mmol·L^− 1^ NaCl; (3) NaCl + Mt: Hoagland nutrient solution plus 150 mmol·L^− 1^ NaCl and 100 mmol·L^− 1^ melatonin

**Fig. 3:**
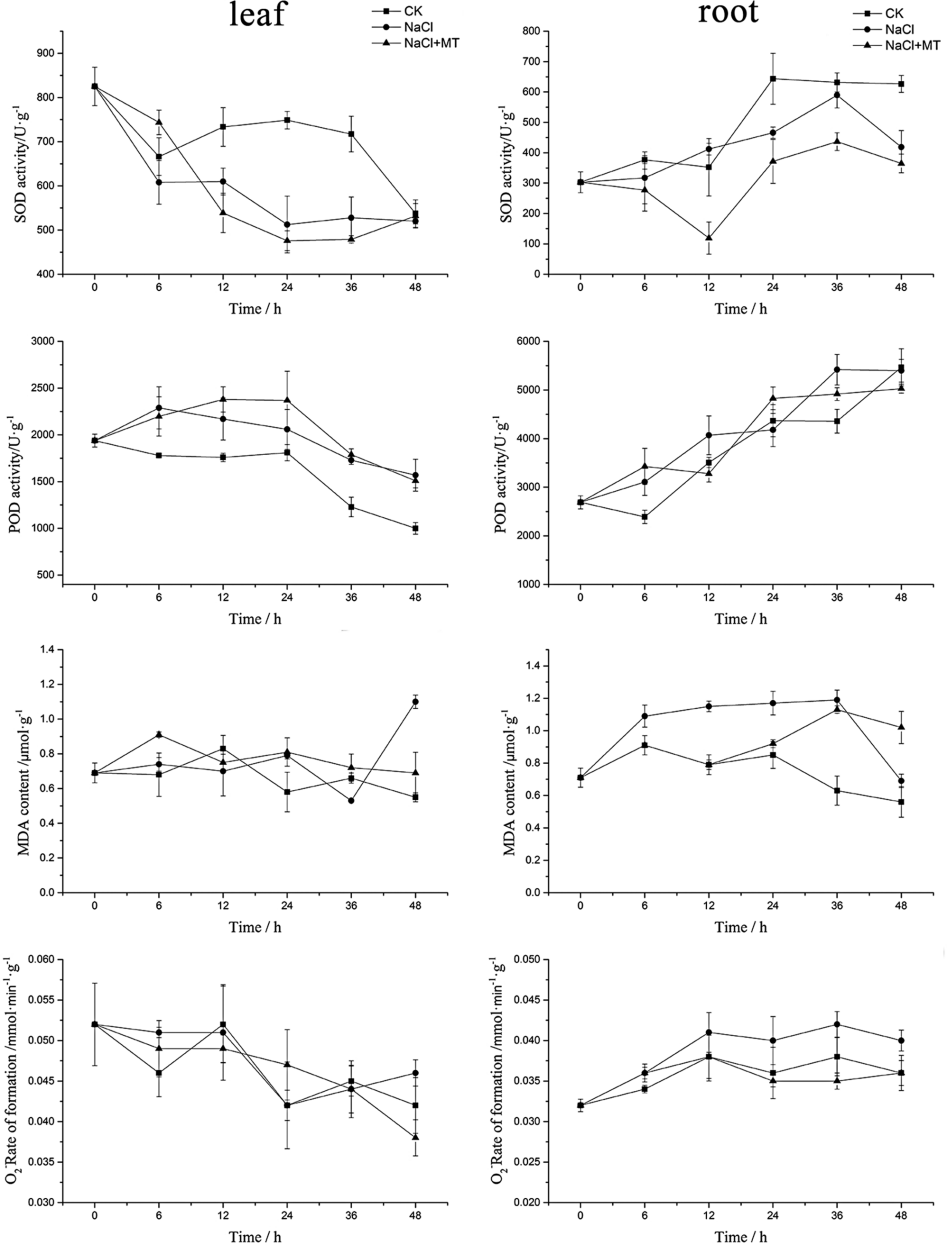
Determination of physiological indexes (SOD, POD, MDA, O^2−^) of two cultivars under NaCl and NaCl + melatonin (MT) treatments on Heihu

After 10 days of salt stress treatment on Heihu, shoot and root growth were found to be significantly inhibited. We found that the growth inhibition of common bean seedlings under NaCl stress was effectively alleviated by adding melatonin (Fig. 2). To further determine the sampling time and sampling tissue appropriate for transcriptome analysis, physiological indexes of experimental materials were dynamically measured under NaCl and NaCl + melatonin treatments, respectively. The results showed that changes in SOD and POD activity, MDA content, and O^2−^ production rate in the leaves over time did not show a clear trend (Fig. 3).

Root parameters showed a certain trend: SOD and POD activity began to increase at 6 h after NaCl treatment, but the difference between different treatment groups at each time point was not significant. MDA content and O^2−^ production rate also showed marked changes in each treatment group at 6–12 h after NaCl treatment. NaCl stress group showed higher MDA content and O^2−^ production rate than the control group, and melatonin effectively reduced the contents of MDA content and O^2−^ production rate to the levels observed in the control, indicating that melatonin alleviates the oxidative damage of root cells induced by NaCl, improves cell vitality, and alleviates NaCl toxicity in common bean. According to the above analysis, it can be determined that the following transcriptome sampling time is 6 h after treatment. The strong correlation between the physiological changes in the root under various treatments indicates that the root responds significantly to NaCl stress. Therefore, the root was determined to be the appropriate sampling tissue for transcriptomics.

### Differences in the metabolite profile between Heihu and F4226

To explore the changes in the metabolite profile under NaCl stress (Heihu and F4226), a metabolomic analysis was performed. The metabolites identified above were assigned to the Kyoto Encyclopedia of Genes and Genomes (KEGG) and PlantCyc databases. In total, 1743 metabolites were classified into 20 level-2 KEGG pathways, 702 of which were assigned to “metabolism.” Under the “metabolism” term, the highest number of metabolites was associated with “Global and overview maps”, followed by “amino acid metabolism,” “biosynthesis of other secondary metabolites,” and “metabolism of cofactors and vitamins” (Additional file 5). Moreover, 475 metabolites were assigned to “metabolic pathways,” 269 and 30 of which were involved in “biosynthesis of other secondary metabolites” and “biosynthesis of plant hormones,” respectively (Additional file 6).

**Fig. 4:**
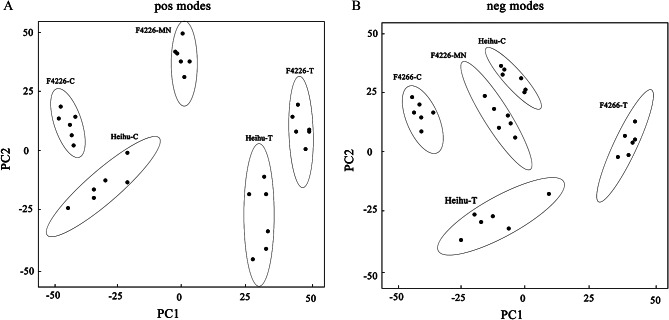
Principal component analysis score plots were derived from metabolite ions acquired from positive (**A**) and negative (**B**) modes

**Fig. 5:**
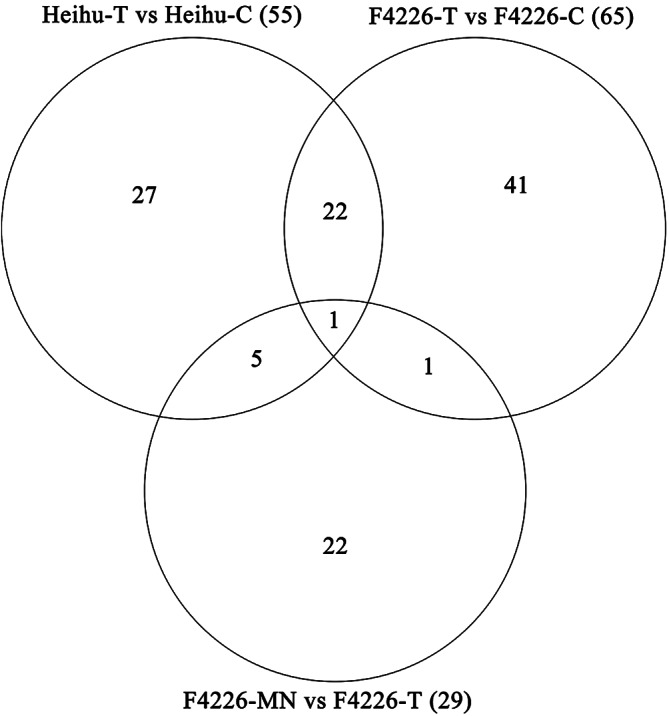
Potential markers were selected by comparing quantitative differences in mass ions in positive (c) and negative (d) modes between Heihu-T and Heihu-C, between F4226-T and F4226-C, and between F4226-MN and F4226-T. *Note*: C: control; T: NaCl treatment; MN: NaCl and melatonin

To compare the metabolite composition under NaCl stress, datasets obtained for five varieties (F4226-C, F4226-T, F4226-MN, Heihu-C, and Heihu-T) using UPLC-Triple-TOF5600plus-MS in the positive and negative ion modes were subjected to PCA. The results showed that the 5 samples were clearly separated in the PC1×PC2 score plots (Fig. 4). Indeed, the five samples were clearly separated along the first principal component (PC1) in positive and negative ion modes. These results were consistent with the source of the samples. The variables responsible for the differences were selected through statistical analysis. A total of 556,529 mass ions were selected between Heihu-T and Heihu-C, between F4226-T and F4226-C, and between F4226-MN and F4226-T, respectively (Fig. 5).

### Identification of differentially expressed genes between Heihu and F4226

**Fig. 6:**
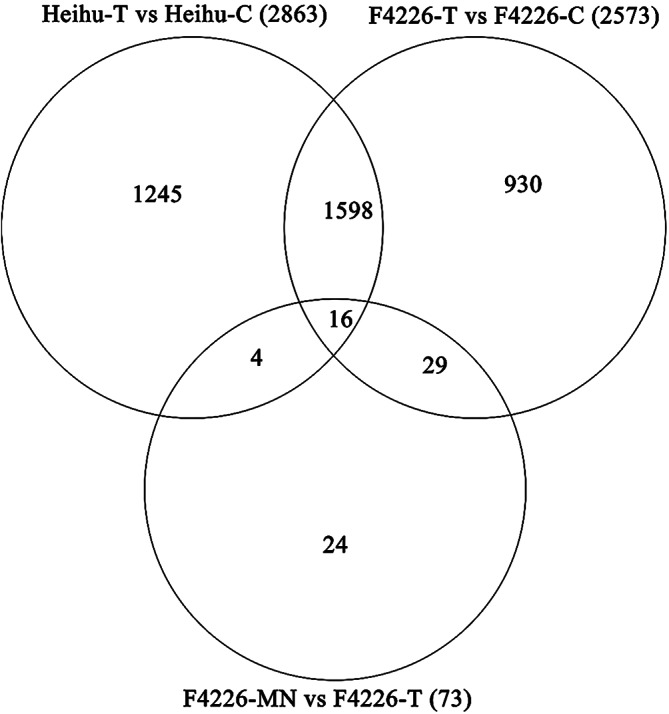
Gene expression reprogramming under salt stress in common bean plant. Venn diagrams displaying the overlap among differentially expressed genes identified in common bean plants. *Note*: C: control; T: NaCl treatment; MN: NaCl and melatonin

Fifteen pooled mRNA samples were used to construct libraries for high-throughput sequencing. The differentially expressed genes (DEGs) were grouped into five major groups (Additional file 7). Among the 3846 significantly upregulated and downregulated genes (Fig. 6), 2863 and 2573 were transiently induced under NaCl stress in Heihu and F4226, respectively (as seen in the Heihu-T vs. Heihu-C and F4226-T vs. F4226-C comparisons), whereas 1614 were common between both comparison groups. The F4226-MN vs. F4226-T comparison showed 73 DEGs, and 16 were common among three comparison groups. The results showed that DEGs were mainly concentrated in the comparisons Heihu-T vs. Heihu-C and F4226-T vs. F4226-C. After functional annotation of the DEGs, we performed gene ontology (GO) classification and a GO term enrichment analysis as the first step toward understanding the molecular changes (Additional files 8 and 9). The enrichment analysis revealed that one of the functions that dominated the NaCl stress was transcription factor activity.

**Fig. 7:**
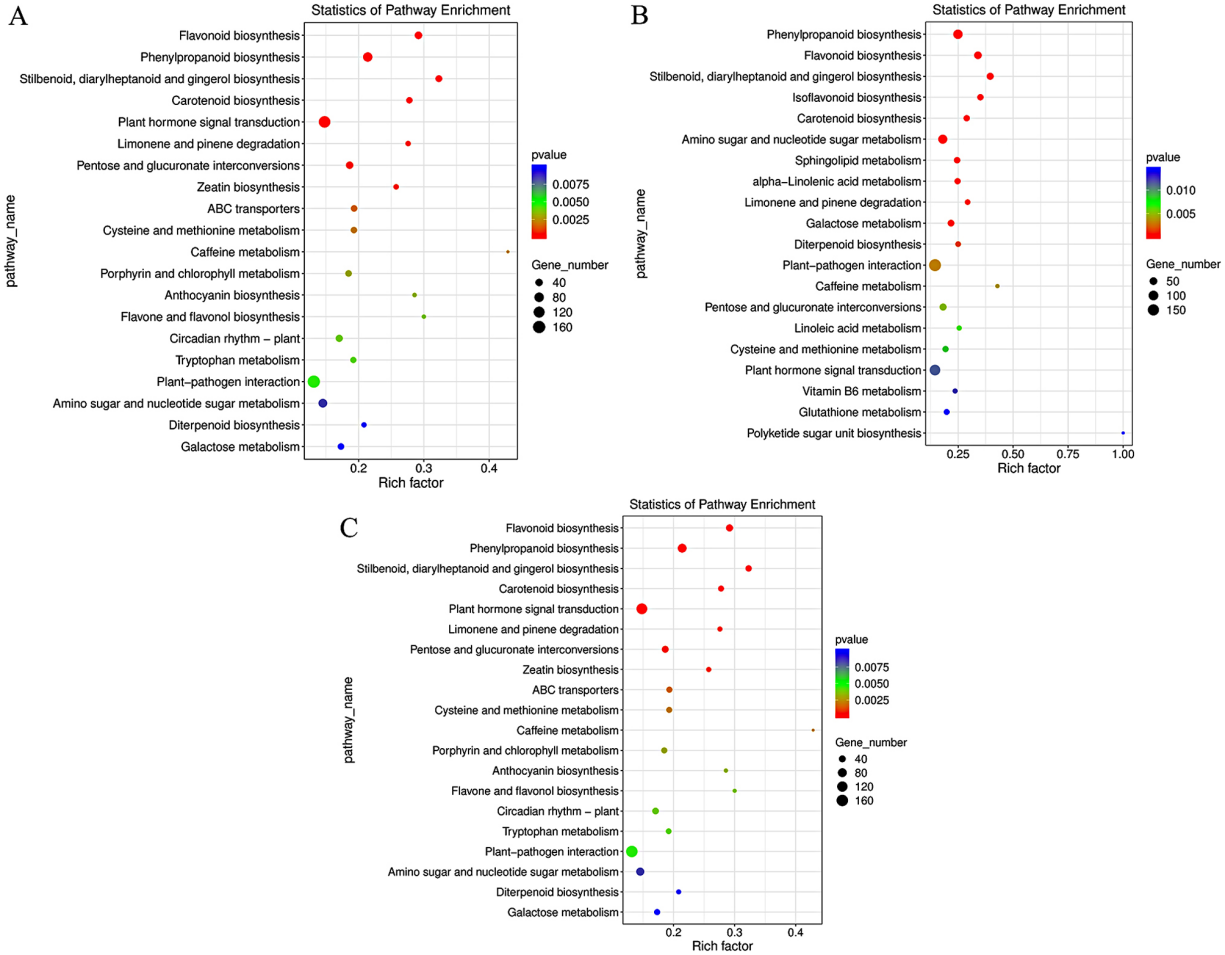
KEGG enrichment analysis of differentially expressed genes in the F4226-T vs F4226-C (**A**), Heihu-T vs Heihu-C (**B**) and F4226-MN vs F4226-T (**C**) groups, and the top 20 KEGG annotated scatter plots were sorted according to enrichment factors

The metabolic pathways enriched in the two common bean varieties under salt stress were sorted, and the top 20 pathways were selected to generate scatter plots. The KEGG pathways ‘flavonoid biosynthesis’ and ‘diterpenoid biosynthesis’ were enriched in both varieties, indicating that both flavonoid metabolism and terpenoid metabolism are involved in the response to salt stress (Fig. 7). The unique metabolic pathways of significant difference in the F4226 correlation comparison group were zeatin biosynthesis, ABC transduction, porphyrin and chlorophyll metabolism, anthocyanin biosynthesis, flavonol and flavonol biosynthesis, plant circadian rhythm, and tryptophan metabolism, Heihu is isoflavone biosynthesis, sphingoid metabolism, isoflavone biosynthesis, sphingoid metabolism, tryptophan metabolism, and Heihu is isoflavone biosynthesis. Alpha-linolenic acid metabolism, linolenic acid metabolism, Vitamin B6 metabolism, glutathione metabolism, and ketogenic glycounit biosynthesis (Fig. 7).

There were 73 DEGs in F4226-MN vs. F4226-T group, and 45 genes were annotated using KEGG pathway enrichment analysis. The most highly enriched pathway was steroid biosynthesis, which was associated with two genes: *Phvul.002G074300* and *Phvul.006G197900* showed the same expression pattern and were highly expressed in Heihu-C, Heihu-T, and F4226-C but were inhibited in F4226-T. Melatonin promotes sterol biosynthesis (Additional file 10). In addition, two genes were enriched in the ABC transporter pathway: *Phvul.001G187800* and *Phvul.001G218000* were differentially expressed only in F4226 under salt stress after melatonin treatment; and the gene *Phvul.001G218000* was annotated as belonging to the ABCB1 subfamily. The specific role of this gene is unclear, but melatonin treatment inhibited its expression (Additional file 11).

Moreover, three metabolic pathways related to terpenoid biosynthesis were enriched in three DEGs: *Phvul.006G197900*, *Phvul.005G111700*, *Phvul.007G228200*. *Phvul.007G228200* was annotated in the metabolic pathway “Biosynthesis of ubiquinone and other terpenoid quinones.” The expression of this gene increased in both varieties under salt stress, and melatonin treatment inhibited the expression. The pathway “biosynthesis of sesquiterpenes and triterpenes” was enriched in *Phvul.006G197900*. Melatonin treatment promoted the expression of *Phvul.005G111700*, the biosynthesis pathway that was enriched in this DEG was not identified (Additional file 10). In addition, three genes enriched in the “phenylpropane biosynthesis” pathway, *Phvul.005g046000*, *Phvul.009g244500*, and *Phvul.011g055800* were also identified. In “tryptophan metabolism,” the gene *Phvul.009G210332* was found to be differentially expressed in all three comparison groups; in “flavonoid metabolism,” the gene *Phvul.005G010000* was upregulated in Heihu-T, and its expression was increased at a low level in F4226-MN (Additional file 11). Melatonin treatment regulates the expression of genes involved in steroid biosynthesis, phenylpropane biosynthesis, terpenoid metabolism, tryptophan metabolism, and flavonoid metabolism and a gene in the ABCB subfamily of ABC transporters.

### Comprehensive analysis of the metabolome and transcriptome

GO enrichment of DEGs in F4226 and Heihu under salt stress showed that cell-wall-related DEGs were enriched in Heihu, but no cell wall-related metabolites were detected in the metabolome. KEGG pathway enrichment analysis of the differentially expressed genes (DEGs) in F4226 and Heihu under salt stress showed that genes related to flavonoid biosynthesis and terpenoid metabolism were enriched in the two varieties. Analysis of differential expression of metabolites (DEMs) also identified solanine (related to terpenoid metabolism) in the two varieties. These findings indicate that both flavonoid metabolism and terpenoid metabolism are involved in the salt stress response of salt-sensitive variety F4226 and salt-tolerant variety Heihu.Isoglycyrrhizin, a metabolite related to flavonoid biosynthesis, was found to be differentially expressed in Heihu. However, no metabolites related to flavonoid metabolism were found to be differentially expressed in F4226. Metabolites related to flavonoid biosynthesis found to be differentially expressed in F4226 were differentially expressed in both varieties under salt stress.

**Fig. 8:**
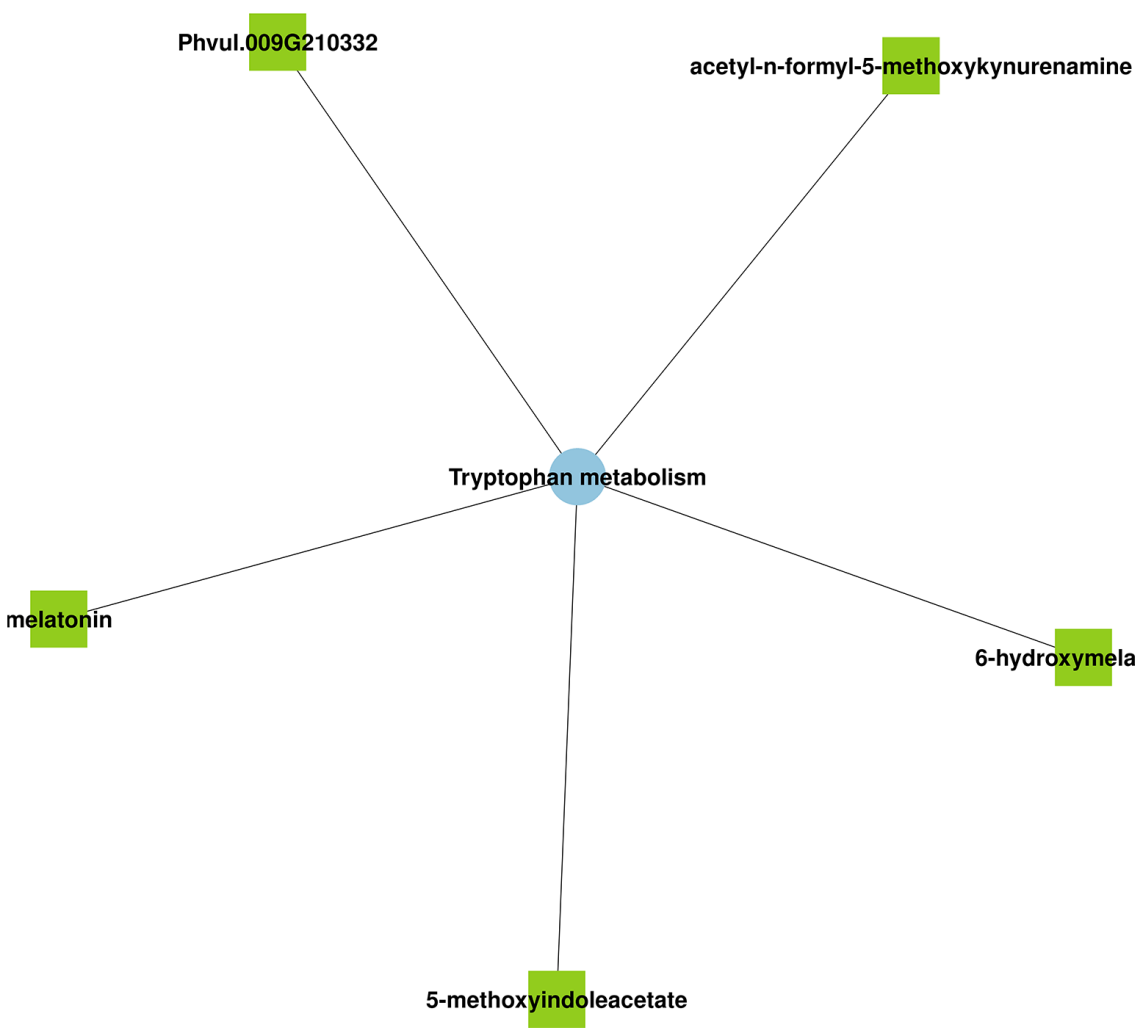
Comprehensive metabolomic and transcriptomic analysis in F4226-MN vs. F4226-T

In F4226 treated with melatonin (F4226-MN vs. F4226-T) under salt stress, melatonin was found to regulate the *Phvul.009G210332* gene and metabolites C05642 (N-acetyl-N-2-formyl-5-methoxycanurine), C05643 (6-hydroxymelatonin), C05660 (5-methoxyindoleacetic acid) involved in the “tryptophan metabolism” pathway. C05642 and C05643 are decomposition products of tryptophan. The findings indicate that exogenous melatonin influenced the metabolic processes in treated common bean plants (Fig. 8). In addition, melatonin was found to regulate one gene associated with the flavonoid metabolism pathway and the isoflavone metabolite C00858 (formononetin) in the phenylpropane biosynthesis pathway, indicating that melatonin affected flavonoid metabolism.

## Discussion

Previous studies have shown that salt stress affects plant cell wall composition [34]. In the present study, GO enrichment analysis shows that the GO terms “cell wall organization” and “plant cell wall” were enriched in several cell wall-related genes only in Heihu. Flavonoids constitute the main group of polyphenolic compounds that play numerous molecular and biochemical roles in plants, such as signaling, free radical scavenging, mediating auxin transport, and plant defense [35]. Flavonoids have long been considered to have health-related functions in the human body, including antioxidant, antimicrobial, and anticancer properties [36]. Flavonoids constitute the main group of plant polyphenolic compounds that play numerous molecular and biochemical roles in plants, such as signaling, free radical scavenging, mediating auxin transport, and plant defense. While salt stress interferes with the initial growth of plants by inducing osmotic stress within the cells, which is alleviated by the accumulation of flavonoids [37]. The KEGG enrichment analysis of the DEGs identified in F4226 and Heihu under salt stress showed that flavonoid-related pathways were enriched in the two varieties. The KEGG enrichment analysis of DEMs between the two varieties under salt stress showed that isolicorice, a metabolite involved in flavonoid biosynthesis, was also significantly enriched in Heihu. Some studies have shown that isolicorice has an inhibitory effect on tumors [38], but its role in salt stress tolerance remains unknown, and it is suggested as a candidate metabolite in tolerance to salt stress in kidney bean. Salt stress has been shown to promote the production of plant terpenoids [39], and some terpenoids play a role in plant tolerance to biotic and abiotic stress [40]. Present findings suggest that terpenoid metabolism in the two varieties changed significantly under salt stress. The KEGG enrichment analysis of DEGs showed that genes related to diterpenoid metabolism were enriched in the two varieties under salt stress. KEGG analysis of DEMs showed that enrichment of solanine related to biosynthesis of terpenoids, polyketones, and alkaloids associated with terpenoid metabolism in both cultivars. In addition, unique metabolic pathways in salt-tolerant varieties for example, alpha-linolenic acid metabolism and linolenic acid metabolism unique to Heihu under salt stress may play a role in tolerance to salt stress. In soybean seedlings under salt stress, the application of linoleic acid was shown to preserve the function of vacuoles [41], and vacuoles play an important role in plant tolerance to salt stress. The DEGs and DEMs associated with these metabolic pathways provide the basis for the discovery of salt-tolerant genes and metabolites in common bean.

Two hypotheses have been proposed for melatonin, suggesting that it acts either as a phytohormone itself or as a master regulator and mediator within various phytohormone networks [42]. Studies have shown that it improves the tolerance of various plants to salt stress [43, 44]. The analysis of DEGs and DEMs identified in the F4226-MN vs. F4226-T comparison showed that after melatonin treatment, the accumulation of melatonin and its two metabolites in salt-stressed F4226 was higher, indicating that melatonin entered the plant tissue and was metabolized. Under salt stress, the cell wall structure is modified to adapt to the new environmental conditions, thus regulating the tolerance of common bean to salt stress. GO enrichment analysis showed that as many as six cell-wall-related pathways were enriched in the F4226-MN vs. F4226-T comparison. The expression levels of seven genes *Phvul.009g186400*, *Phvul.009G244500*, *Phvul.002G152900*, *Phvul.003G213800*, *Phvul.005G046000*, *Phvul.006G147600*, and *Phvul.006g147500* in F4226 after melatonin treatment were similar to those in the salt-tolerant variety Heihu. These cell-wall-related DEGs may play a key role in tolerance to salt stress in common bean. Moreover, the GO term “sodium ion transmembrane transport” was also enriched in DEGs, including Phvul.004G177300. The expression levels of *Phvul.004G177300* in salt-treated F4226 after melatonin treatment were similar to those in salt-stress-treated Heihu. Under salt stress, plants regulate intracellular and intracellular osmotic potential through ion transmembrane transport. Changes in the expression level of this gene affects Na^+^ expulsion and transport to vacuoles and play an important role in plant tolerance to salt stress. In the present study, KEGG analysis of the DEGs in the F4226-MN vs. F4226-T comparison showed that one gene involved in the flavonoid biosynthesis pathway was differentially expressed. Melatonin further regulates flavonoid biosynthesis by regulating the expression of this gene. Furthermore, the accumulation of C00858 (formononetin), an isoflavone metabolite, increased. Studies have shown that formononetin improves potato yield by promoting the activity of potato mycorrhizal fungi [45], and salt-tolerant *Rhizobium* strains improve the salt stress tolerance of bean [2]. Formononetin may enhance tolerance to salt stress by promoting the growth of salt-tolerant *Rhizobium*. Terpenes are induced by biotic and abiotic stressors; abiotic stress has been shown to induce terpene production in various plants such as grape [46] and tea tree [47]. In the present study, KEGG analysis of DEGs in the F4226-MN vs. F4226-T comparison showed that various terpene biosynthetic pathways were also enriched. This finding indicates that melatonin treatment regulated terpene synthesis under salt stress, and terpene synthesis plays a role in plant tolerance to high salt conditions. Among all the DEGs related to terpene biosynthesis, *Phvul.007g228200* was upregulated in Heihu and F4226 under salt stress. However, the expression level of this gene in F4226 under salt stress was significantly higher than that in Heihu under salt stress. Melatonin treatment reduced the expression level of this gene in F4226 under salt stress, indicating that melatonin plays an important role in tolerance to salt stress in string bean. In conclusion, melatonin might enhance the tolerance to salt stress by affecting genes and metabolites in these metabolic pathways in common bean. This study provides a preliminary understanding of the molecular mechanism and pathway of response to salt stress in common bean and provides a basis for integrating salt tolerance genes to improve the salt tolerance of common bean in future studies.

## Conclusions

**Fig. 9:**
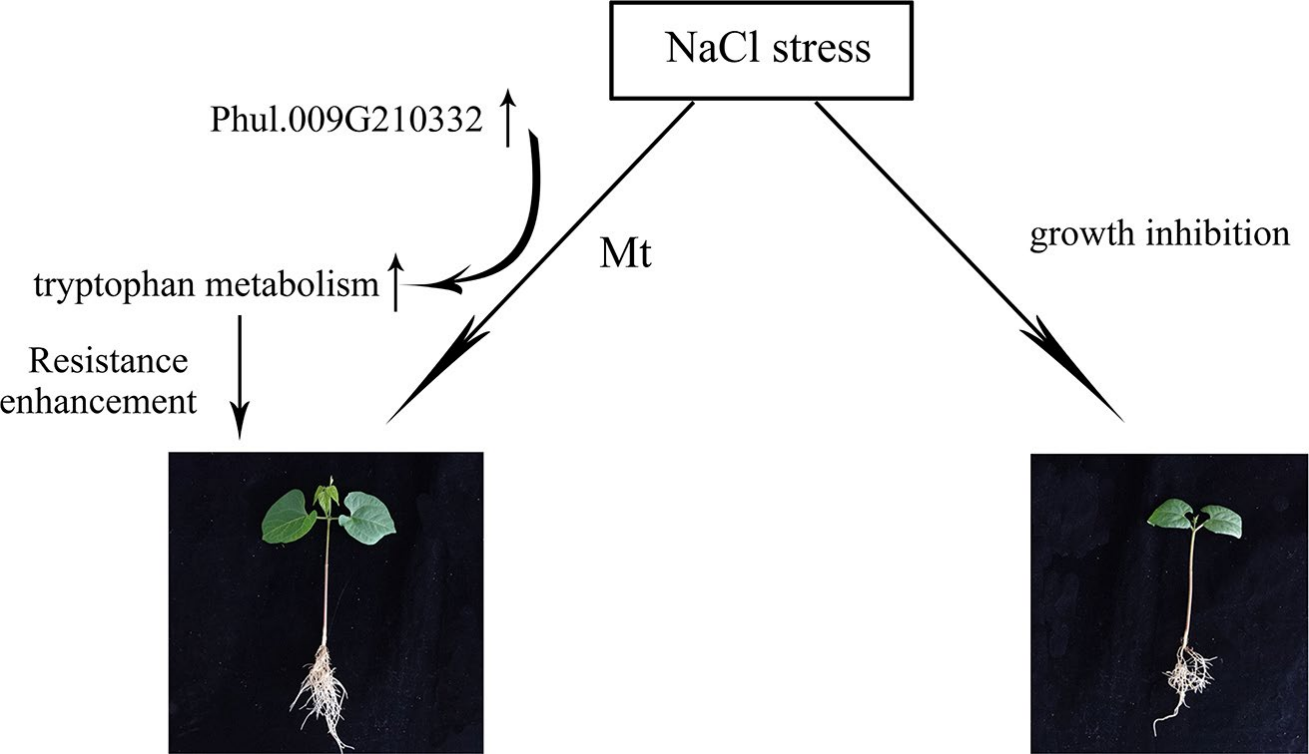
Effect mechanism of melatonin in common bean under NaCl stress

In this study, using metabolomic and transcriptomic analyses, DEGs and DEMs in the salt-sensitive cultivar F4226 and the salt-tolerant cultivar Heihu were identified following salt stress treatment. The transcriptomic and metabolomic data suggested that F4226 and Heihu have similar salt stress responses; however, some differences do exist. Melatonin regulates the gene Phvul.009G210332 and metabolites C05642 (N-acetyl-N-2-formyl-5-methoxycanurine), C05643 (6-hydroxymelatonin), and C05660 (5-methoxyindoleacetic acid) involved in the “tryptophan metabolism” pathway. The metabolites C05642 and C05643 are decomposition products of tryptophan, which indicates that melatonin entered the plant tissue and was metabolized. Melatonin promote the synthesis and metabolism of tryptophan, which is crucial to plant metabolism, growth, maintenance, and repair (Fig. 9). This study provides a preliminary understanding of the molecular mechanism and pathway of response to salt stress in common bean and provides a basis for integrating salt tolerance genes to improve the salt tolerance of common bean in future studies.

### Electronic supplementary material

Below is the link to the electronic supplementary material.

**Supplementary Material 1:** List of cultivars and growth habit in this study

## Data Availability

Transcriptome data have been uploaded to the GEO database: https://www.ncbi.nlm.nih.Gov/geo/, Registration number is: GSE192891.
