# Supplementary material for: Possible melatonin-induced salt stress tolerance pathway in Phaseolus vulgaris L. using transcriptomic and metabolomic analyses
Source: BMC Plant Biol. 2024 Jan 25;24:72. doi: 10.1186/s12870-023-04705-x (PMC10809447; doi:10.1186/s12870-023-04705-x)
Supplement: Supplementary file 1 — Supplementary Material 1: List of cultivars and growth habit in this study [file 12870_2023_4705_MOESM1_ESM.docx]

**Supplementary Materials**

Additional file 1 List of cultivars and growth habit in this study

| code | cultivars | growth habit |
| --- | --- | --- |
| 1 | Hong jin gou | indeterminate viny |
| 2 | Xiang jiao you dou | indeterminate viny |
| 3 | Sheng feir | indeterminate viny |
| 4 | Qi ji | indeterminate viny |
| 5 | Zao feng | indeterminate viny |
| 6 | Kuai jian | indeterminate viny |
| 7 | Da zi pao | indeterminate viny |
| 8 | Di dou | determinate brush |
| 9 | Jiu yue | determinate brush |
| 10 | Jia you dou | determinate brush |
| 11 | Yi ke song | determinate brush |
| 12 | Dan se qiu | determinate brush |
| 13 | Aug-73 | determinate brush |
| 14 | May-97 | indeterminate viny |
| 15 | 923 | indeterminate viny |
| 16 | Te nen | indeterminate viny |
| 17 | Tai man | indeterminate viny |
| 18 | Wu jin 6 | indeterminate viny |
| 19 | F0119 | indeterminate viny |
| 20 | F0678 | determinate brush |
| 21 | F0705 | determinate brush |
| 22 | F1736 | determinate brush |
| 23 | F2320 | determinate brush |
| 24 | F3420 | indeterminate viny |
| 25 | F4150 | indeterminate viny |
| 26 | F4226 | indeterminate viny |
| 27 | BE221-1 | determinate brush |
| 28 | Shuang feng NO.3 | indeterminate viny |
| 29 | D6 | determinate brush |
| 30 | D8 | determinate brush |
| 31 | D10 | determinate brush |
| 32 | D11 | determinate brush |
| 33 | D13 | determinate brush |
| 34 | Zao wu jin | indeterminate viny |
| 35 | Chao shang si ji dou | indeterminate viny |
| 36 | Shi li chang | indeterminate viny |
| 37 | Da ma zhang | indeterminate viny |
| 38 | 851-923-9 | indeterminate viny |
| 39 | May-97 | indeterminate viny |
| 40 | Yin bai tiao | indeterminate viny |
| 41 | Zao man jia | indeterminate viny |
| 42 | Qing feng di dou | determinate brush |
| 43 | Qing dao dou | determinate brush |
| 44 | Jun-81 | determinate brush |
| 45 | Shuang sheng jia dou | determinate brush |
| 46 | Tian ma di dou | determinate brush |
| 47 | Shuang sheng di dou | determinate brush |
| 48 | 5991（81-6） | determinate brush |
| 49 | No. 16 | indeterminate viny |
| 50 | 4月10日 | indeterminate viny |
| 51 | Tu zi fan bai yan | indeterminate viny |
| 52 | 1996/9/17 | indeterminate viny |
| 53 | 96-8-50 | indeterminate viny |
| 54 | 1996/8/14 | indeterminate viny |
| 55 | 1996/8/7 | indeterminate viny |
| 56 | 1996/8/4 | indeterminate viny |
| 57 | 1996/8/2 | indeterminate viny |
| 58 | Chao chang si ji dou | indeterminate viny |
| 59 | San dao mei | indeterminate viny |
| 60 | Da you dou | indeterminate viny |
| 61 | Hei huang dou | indeterminate viny |
| 62 | Ya ta jia | indeterminate viny |
| 63 | Ji dou jiao | indeterminate viny |
| 64 | Ha cai dou NO. 8 | indeterminate viny |
| 65 | Di you dou | determinate brush |
| 66 | You dou | indeterminate viny |
| 67 | Chao chang jia dou | indeterminate viny |
| 68 | Ju feng | indeterminate viny |
| 69 | Bo li cui | indeterminate viny |
| 70 | 2504Jia dou | indeterminate viny |
| 71 | Lv long dou | indeterminate viny |
| 72 | Ha cai dou NO.1 | indeterminate viny |
| 73 | Jin long | indeterminate viny |
| 74 | Yi chuan feng | indeterminate viny |
| 75 | Fan bai yan | indeterminate viny |
| 76 | Zi hua you dou | indeterminate viny |
| 77 | Yu dou | indeterminate viny |
| 78 | Mar-78 | indeterminate viny |
| 79 | Mao yan | indeterminate viny |
| 80 | Lian pu | indeterminate viny |
| 81 | Renaya | determinate brush |
| 82 | A18 | determinate brush |
| 83 | P18039 | determinate brush |
| 84 | P16071 | determinate brush |
| 85 | P16133 | indeterminate viny |
| 86 | P16152 | indeterminate viny |
| 87 | P16158 | indeterminate viny |
| 88 | P16157 | indeterminate viny |
| 89 | P16156 | determinate brush |
| 90 | P16079 | determinate brush |
| 91 | P18017 | indeterminate viny |
| 92 | P18009 | indeterminate viny |
| 93 | P18084 | indeterminate viny |
| 94 | P19011 | indeterminate viny |
| 95 | P19002 | indeterminate viny |
| 96 | P18051 | indeterminate viny |
| 97 | P19030 | indeterminate viny |
| 98 | P16009 | indeterminate viny |
| 99 | P19024 | determinate brush |
| 100 | P16024-8 | determinate brush |
| 101 | Hacaidou | determinate brush |
| 102 | P18016 | determinate brush |
| 103 | A24-4 | determinate brush |
| 104 | P16038 | determinate brush |
| 105 | Hayousan | indeterminate viny |
| 106 | P18041 | indeterminate viny |
| 107 | P16203-16 | indeterminate viny |
| 108 | P16030 | indeterminate viny |
| 109 | P18012 | determinate brush |
| 110 | P16314 | determinate brush |
| 111 | P19023 | indeterminate viny |
| 112 | P19006 | determinate brush |
| 113 | P16070 | determinate brush |
| 114 | P18045 | determinate brush |
| 115 | P18037 | determinate brush |
| 116 | P18002 | determinate brush |
| 117 | P16028 | indeterminate viny |
| 118 | P16333 | indeterminate viny |
| 119 | P18014 | indeterminate viny |
| 120 | P18069 | indeterminate viny |

Additional file 2 Basic growth index of material

| Variety | Relative growth | Variable coefficient | Relative root-shoot ratio | Variable coefficient | Relative plant height | Variable coefficient | Relative leaf area | Variable coefficient | Relative net photosynthesis | Variable coefficient |
| --- | --- | --- | --- | --- | --- | --- | --- | --- | --- | --- |
| Renaya | 0.18 | 69.11% | 1.33 | 14.32% | 0.55 | 29.22% | 0.37 | 45.93% | 0.59 | 25.57% |
| A18 | 0.09 | 83.55% | 0.73 | 15.52% | 0.68 | 18.78% | 0.37 | 45.62% | 0.20 | 66.41% |
| P18039 | 0.31 | 53.21% | 1.34 | 14.41% | 0.78 | 12.59% | 0.56 | 28.54% | 0.27 | 57.02% |
| P16071 | 0.03 | 94.72% | 0.70 | 17.73% | 0.91 | 4.81% | 0.61 | 24.12% | 0.32 | 50.99% |
| P16133 | 0.10 | 81.97% | 0.80 | 11.34% | 0.77 | 13.11% | 0.44 | 39.27% | 0.36 | 46.54% |
| P16152 | 0.11 | 79.40% | 0.77 | 12.73% | 1.59 | 22.74% | 0.58 | 26.55% | 0.31 | 52.43% |
| P16158 | -0.23 | 158.82% | 0.73 | 15.35% | 0.68 | 18.99% | 0.36 | 46.88% | 0.26 | 59.16% |
| P16157 | 0.26 | 58.31% | 0.87 | 7.13% | 0.76 | 13.49% | 0.54 | 29.47% | 0.32 | 51.43% |
| P16156 | 0.21 | 65.92% | 0.93 | 3.81% | 0.96 | 2.24% | 0.60 | 24.93% | 0.35 | 47.91% |
| P16079 | 0.44 | 39.23% | 1.09 | 4.39% | 0.55 | 29.00% | 0.71 | 16.74% | 0.44 | 39.30% |
| P18017 | -0.32 | 192.80% | 1.30 | 13.09% | 0.54 | 29.71% | 0.46 | 37.02% | 0.69 | 18.55% |
| P18009 | 0.42 | 41.08% | 1.24 | 10.76% | 0.88 | 6.58% | 0.54 | 29.51% | 0.69 | 18.31% |
| P18084 | 0.10 | 81.18% | 0.96 | 1.80% | 0.58 | 26.38% | 0.43 | 40.07% | 0.41 | 42.21% |
| P19011 | 0.06 | 89.12% | 1.13 | 6.32% | 0.55 | 29.31% | 0.41 | 42.34% | 0.26 | 58.81% |
| P19002 | 0.11 | 79.75% | 0.92 | 4.31% | 0.60 | 25.00% | 0.90 | 5.41% | 0.27 | 57.32% |
| P18051 | 0.27 | 57.75% | 1.75 | 27.19% | 0.46 | 36.77% | 0.48 | 35.59% | 0.86 | 7.53% |
| P19030 | -0.05 | 110.66% | 0.85 | 8.27% | 0.61 | 23.90% | 0.26 | 58.14% | 0.25 | 60.57% |
| P16009 | 0.23 | 62.78% | 0.97 | 1.31% | 0.59 | 25.94% | 0.45 | 37.81% | 0.56 | 27.80% |
| P19024 | 0.16 | 71.95% | 1.23 | 10.40% | 0.79 | 11.51% | 0.39 | 44.17% | 0.30 | 54.16% |
| P16024-8 | 0.15 | 74.46% | 1.34 | 14.48% | 0.90 | 5.31% | 0.42 | 40.55% | 0.28 | 56.10% |
| Hacaidou | -0.03 | 106.25% | 0.88 | 6.29% | 0.63 | 22.43% | 0.35 | 48.29% | 0.33 | 50.10% |
| P18016 | 0.10 | 82.55% | 1.19 | 8.80% | 0.50 | 33.10% | 0.41 | 41.72% | 0.31 | 53.24% |
| A24-4 | 0.13 | 76.25% | 1.53 | 21.04% | 0.63 | 22.39% | 0.31 | 52.22% | 0.41 | 42.24% |
| P16038 | 0.19 | 67.99% | 1.18 | 8.19% | 0.81 | 10.38% | 0.39 | 44.02% | 0.44 | 38.58% |
| Hayousan | 0.44 | 38.43% | 1.04 | 1.93% | 0.78 | 12.45% | 0.41 | 41.75% | 0.47 | 36.07% |
| P18041 | -0.19 | 148.06% | 0.97 | 1.67% | 0.69 | 18.37% | 0.37 | 45.70% | 0.27 | 57.69% |
| P16203-16 | 0.17 | 70.97% | 1.00 | 0.04% | 0.66 | 20.70% | 0.42 | 40.75% | 0.61 | 24.17% |
| P16030 | 0.58 | 26.29% | 1.04 | 1.78% | 0.60 | 24.86% | 0.43 | 39.90% | 0.50 | 33.53% |
| P18012 | 0.62 | 23.52% | 1.15 | 6.76% | 0.82 | 10.12% | 0.70 | 17.71% | 0.60 | 25.04% |
| P16314 | 0.27 | 57.79% | 1.00 | 0.15% | 0.83 | 9.40% | 0.67 | 19.78% | 0.10 | 81.51% |
| P19023 | 0.44 | 38.46% | 1.05 | 2.59% | 0.98 | 1.06% | 0.64 | 21.84% | 0.51 | 32.22% |
| P19006 | -0.68 | 522.22% | 0.98 | 0.97% | 1.04 | 1.75% | 0.29 | 54.67% | 0.06 | 88.37% |
| P16070 | -0.05 | 110.67% | 0.99 | 0.32% | 0.71 | 17.09% | 0.28 | 56.31% | 0.31 | 52.23% |
| P18045 | -1.87 | -328.69% | 1.12 | 5.68% | 0.83 | 9.50% | 0.28 | 56.44% | 0.45 | 37.56% |
| P18037 | -0.46 | 273.47% | 0.67 | 19.50% | 0.77 | 13.30% | 0.52 | 31.41% | 0.44 | 38.89% |
| P18002 | 0.27 | 57.23% | 0.77 | 12.75% | 0.76 | 13.72% | 0.44 | 38.64% | 0.58 | 26.84% |
| P16028 | -0.67 | 500.00% | 0.82 | 9.77% | 0.85 | 7.90% | 0.32 | 50.97% | 0.10 | 81.90% |
| P16333 | -0.64 | 461.02% | 0.74 | 15.23% | 0.53 | 30.78% | 0.54 | 29.85% | 0.21 | 64.98% |
| P18014 | 0.27 | 56.90% | 1.04 | 1.74% | 0.59 | 25.96% | 0.67 | 19.73% | 0.45 | 38.15% |
| P18069 | -0.07 | 114.92% | 0.93 | 3.54% | 0.92 | 4.43% | 0.57 | 27.33% | 0.36 | 46.96% |
| Maximum value | 0.62 | 522.22% | 1.75 | 27.19% | 1.59 | 36.77% | 0.90 | 58.14% | 0.86 | 88.37% |
| Minimum value | -1.87 | -328.69% | 0.67 | 0.04% | 0.46 | 1.06% | 0.26 | 5.41% | 0.06 | 7.53% |
| Mean value | 0.04 | 103.75% | 1.03 | 8.59% | 0.74 | 17.38% | 0.47 | 36.94% | 0.39 | 46.21% |
| Standard deviation | 0.43 | 1.36 | 0.23 | 0.07 | 0.20 | 0.10 | 0.14 | 0.12 | 0.17 | 0.17 |

Additional file 3 Weight of each indicator

| Index | µ Relative growth | µ Relative root-shoot ratio | µ Relative plant height | µ Relative leaf area | µ Relative net photosynthesis |
| --- | --- | --- | --- | --- | --- |
| Mean value | 0.69 | 0.32 | 0.25 | 0.30 | 0.41 |
| Standard deviation | 0.16 | 0.22 | 0.17 | 0.20 | 0.21 |
| CV/% | 0.23 | 0.68 | 0.71 | 0.67 | 0.52 |
| weight /W | 0.08 | 0.24 | 0.25 | 0.24 | 0.18 |

Additional file 4 Correlation between each indicator and D value

|  | Relative growth | Relative root-shoot ratio | Relative plant height | Relative leaf area | Relative net photosynthesis | D值 |
| --- | --- | --- | --- | --- | --- | --- |
| Relative growth | 1 |  |  |  |  |  |
| Relative root-shoot ratio | 0.213 | 1 |  |  |  |  |
| Relative plant height | -0.062 | -0.275 | 1 |  |  |  |
| Relative leaf area | 0.416^**^ | -0.087 | 0.129 | 1 |  |  |
| Relative net photosynthesis | 0.304 | 0.480^**^ | -0.247 | 0.117 | 1 |  |
| D值 | 0.527^**^ | 0.578^**^ | 0.252 | 0.581^**^ | 0.627^**^ | 1 |

Note: * Significant，** [Extremely](file:///D:\\%E5%BA%94%E7%94%A8%E7%A8%8B%E5%BA%8F\\%E6%9C%89%E9%81%93\\Dict\\7.5.2.0\\resultui\\dict\\?keyword=extremely)[significant](file:///D:\\%E5%BA%94%E7%94%A8%E7%A8%8B%E5%BA%8F\\%E6%9C%89%E9%81%93\\Dict\\7.5.2.0\\resultui\\dict\\?keyword=significant)





Additional file 5 KEGG pathway classification (The x-axis represents level-2 terms of the KEGG pathway and the y-axis represents the number of metabolites)


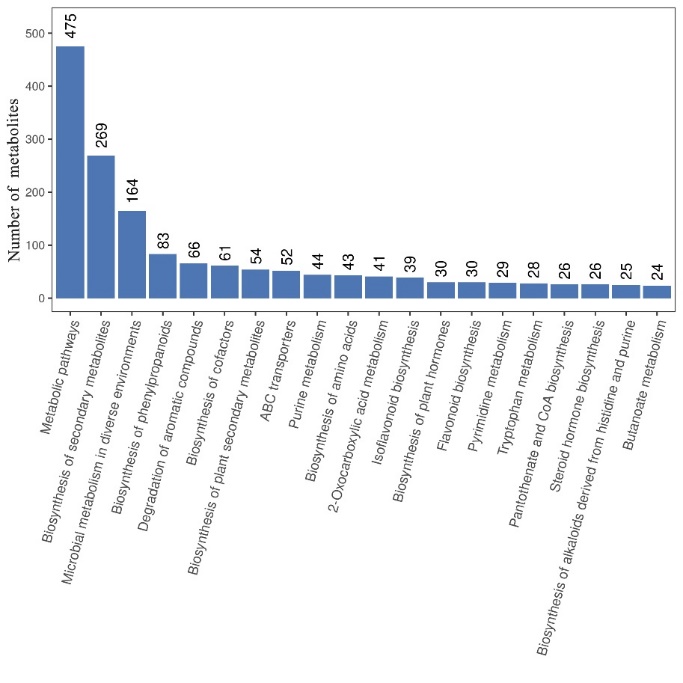


Additional file 6 Identified metabolites classified into the top 20 KEGG pathways. The x-axis represents the top 20 KEGG pathways and the y-axis represents number of identified metabolites involved in this pathway.


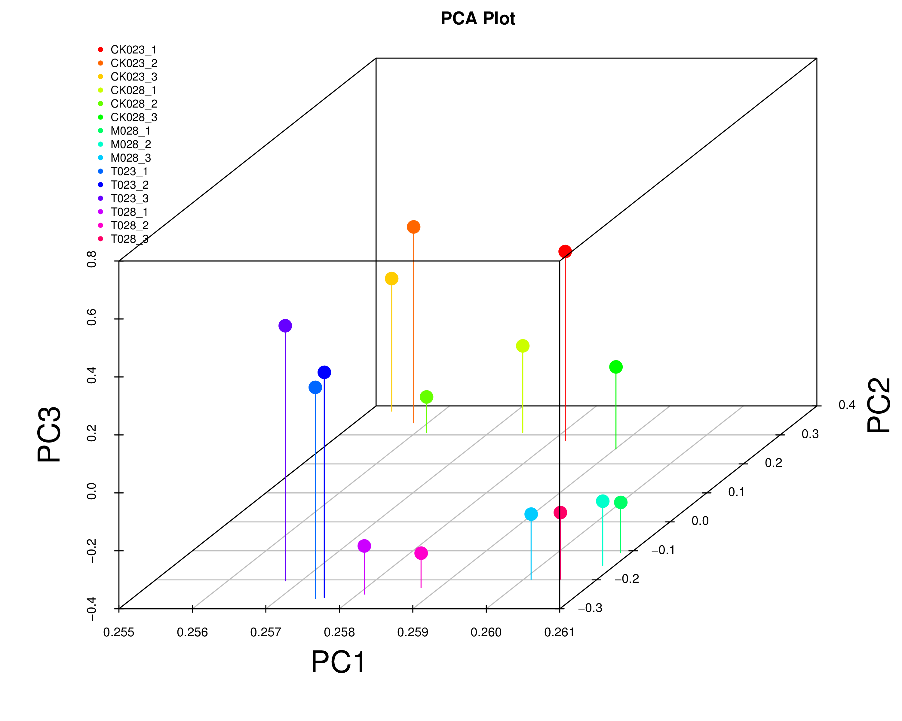


Additional file 7 PCA analysis among samples (PCA score plots were derived from DEGs )


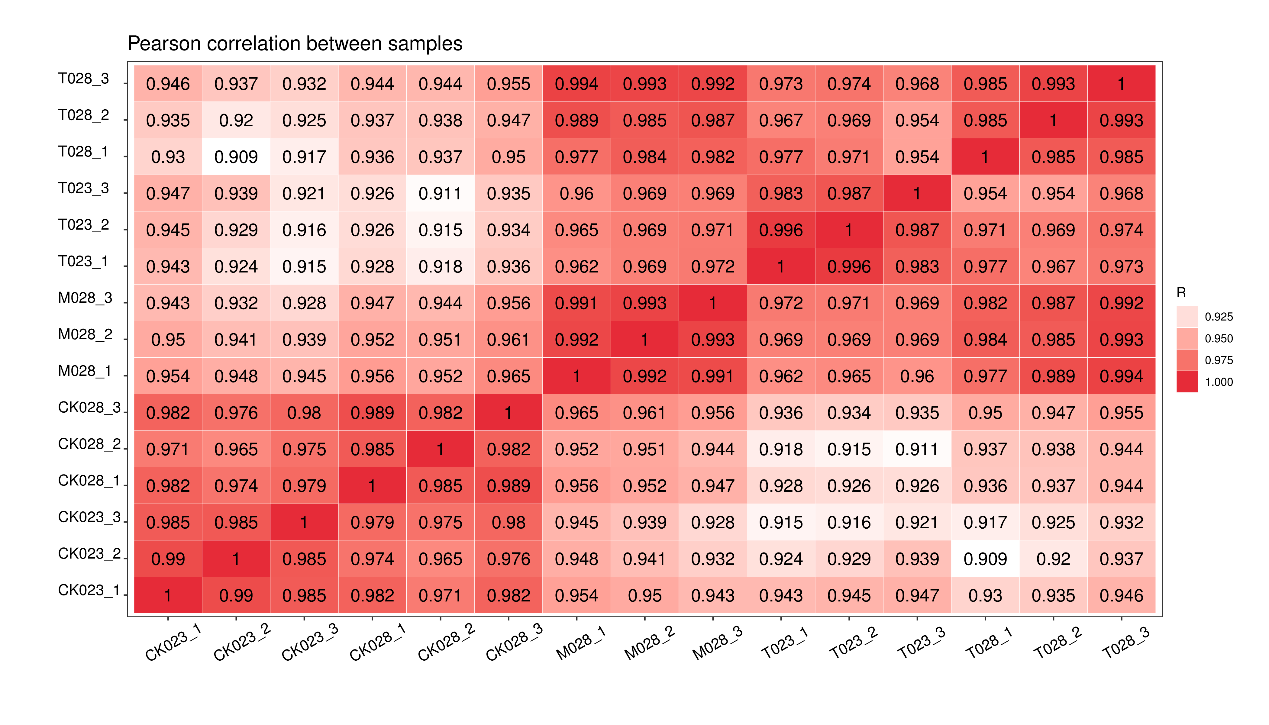


Additional file 8 Pearson correlation coefficients among samples (FPKM expression was used for correlation coefficients)

A B


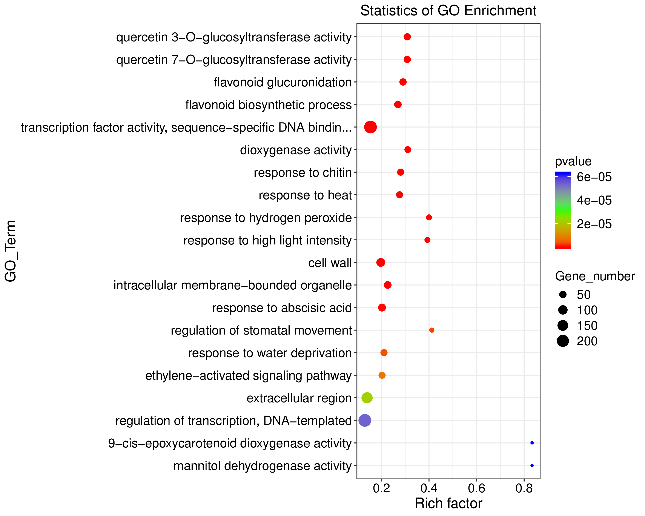

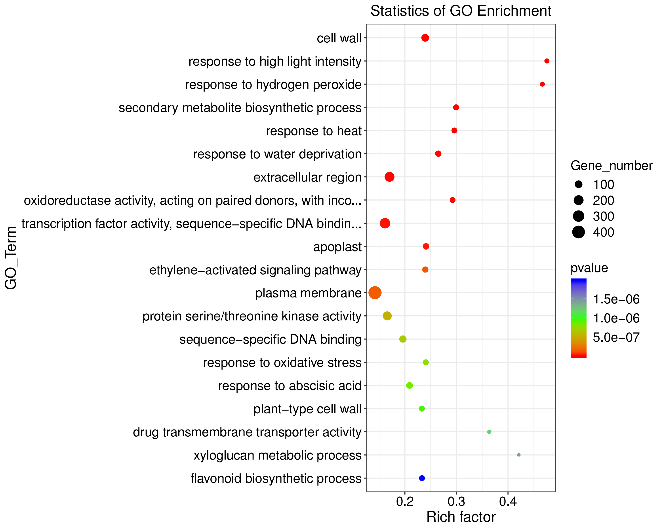


C


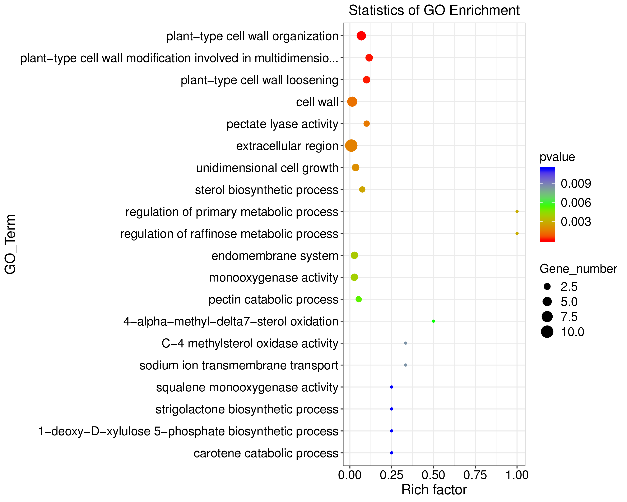


Additional file 9 Selected gene ontology (GO) terms enriched among DEGs identified in common bean plant (A: Heihu-T vs Heihu-C, B: F4226-T vs F4226-C, C: F4226-MN vs F4226-T).

Additional file 10 Key gene expression levels of salt stress resistance in Common bean in KEGG pathway

Additional file 11 Expression levels of key genes resistant to salt stress in common bean in KEGG pathway
